# Variegation of autism related traits across seven neurogenetic disorders

**DOI:** 10.1038/s41398-022-01895-0

**Published:** 2022-04-07

**Authors:** Nancy Raitano Lee, Xin Niu, Fengqing Zhang, Liv S. Clasen, Beth A. Kozel, Ann C. M. Smith, Gregory L. Wallace, Armin Raznahan

**Affiliations:** 1 Department of Psychological and Brain Sciences, Drexel University, Philadelphia, PA 19104 USA; 2 Section on Developmental Neurogenomics, Human Genetics Branch, Division of Intramural Research at the National Institute of Mental Health, National Institutes of Health, Bethesda, MD 20892 USA; 3 Translational Vascular Medicine Branch, Division of Intramural Research at the National Heart, Lung, and Blood Institute, National Institutes of Health, Bethesda, MD 20892 USA; 4 Office of the Clinical Director, Division of Intramural Research at the National Human Genome Research Institute, National Institutes of Health, Bethesda, MD 20892 USA; 5 Department of Speech, Language, and Hearing Sciences, The George Washington University, Washington, DC 20052 USA

**Keywords:** Clinical genetics, Clinical genetics, Psychiatric disorders

## Abstract

Gene dosage disorders (GDDs) constitute a major class of genetic risks for psychopathology, but there is considerable debate regarding the extent to which different GDDs induce different psychopathology profiles. The current research speaks to this debate by compiling and analyzing dimensional measures of several autism-related traits (ARTs) across seven diverse GDDs. The sample included 350 individuals with one of 7 GDDs, as well as reference idiopathic autism spectrum disorder (ASD; *n* = 74) and typically developing control (TD; *n* = 171) groups. The GDDs were: Down, Williams–Beuren, and Smith–Magenis (DS, WS, SMS) syndromes, and varying sex chromosome aneuploidies (“plusX”, “plusXX”, “plusY”, “plusXY”). The Social Responsiveness Scale (SRS-2) was used to measure ARTs at different levels of granularity—item, subscale, and total. General linear models were used to examine ART profiles in GDDs, and machine learning was used to predict genotype from SRS-2 subscales and items. These analyses were completed with and without covariation for cognitive impairment. Twelve of all possible 21 pairwise GDD group contrasts showed significantly different ART profiles (7/21 when co-varying for IQ, all Bonferroni-corrected). Prominent GDD–ART associations in post hoc analyses included relatively preserved social motivation in WS and relatively low levels of repetitive behaviors in plusX. Machine learning revealed that GDD group could be predicted with plausible accuracy (~60–80%) even after controlling for IQ. GDD effects on ARTs are influenced by GDD subtype and ART dimension. This observation has consequences for mechanistic, clinical, and translational aspects of psychiatric neurogenetics.

## Introduction

Advances in psychiatric genetics have led to the identification of a growing number of individually rare, but collectively common genetic variants that are highly penetrant for psychiatric morbidity [1, 2]. Of these, recurrent gene dosage disorders (GDD), including aneuploidies and sub-chromosomal copy number variations (CNVs), have been most amenable to clinical characterization because their incidence yields cohorts that are sufficiently large for group phenotyping [3]. Despite increasing reports on such cohorts, there have been only a few studies which compare behavioral profiles across several GDDs [4, 5]. Such research is important for determining if different genetic lesions induce unique psychopathology profiles. If significant behavioral variegation is observed across GDDs, genetic diagnosis may be used to tailor assessments and improve prognostication. Conversely, weak variegation implies the existence of common biological pathways that “concentrate” different genetic risks, with potentially positive implications for treatment generalizability.

The degree to which different genetic diagnoses impart unique behavioral profiles is hotly-debated [6]. The current study engages with this debate by providing an analysis of variation in autism-related traits (ARTs) across seven GDDs—Down, Williams–Beuren, Smith–Magenis, and several sex chromosome aneuploidy syndromes—using the Social Responsiveness Scale—Second Edition (SRS-2 [7]), a well-validated and widely used questionnaire of ARTs. These seven clinical cohorts enabled analysis of available SRS-2 data across an informatively diverse set of GDDs which varied substantially in their genomic basis (duplications and deletions, aneuploidies, and CNVs) and clinical characteristics (e.g., severity of behavioral disturbance and intellectual disability). Our focus on ARTs rather than diagnostic status was motivated by several considerations. First, autism-related social communication and behavioral flexibility impairments exist as a continuous distribution within the general population [8]. Moreover, these traits are not only elevated in individuals with ASD, but also in groups with diverse non-ASD diagnoses [9–11]. Moreover, variation in ARTs in both the general population and groups with non-ASD psychiatric diagnoses is known to correlate with adaptive functioning and other clinical outcomes [12–15]. Therefore, the degree to which ARTs show dissociable alterations across different genetic disorders carries broad relevance. Second, ARTs provide a powerful context to test for patterned effects of rare genetic disorders on psychopathology, because there is already evidence for their genetic dissociability from quantitative genetic research in population-based samples [16, 17]. Finally, variation in ARTs is linked to variation in cognitive ability at genetic [18–20] and clinical [21] levels. This property makes ARTs particularly well-suited for testing whether apparent differences in psychopathology across genetic disorders are amplified or diminished by the degree of co-occurring cognitive impairment. Thus, the current research sought to examine (a) whether ART profiles, as measured by the SRS-2 subscales, vary as a function of GDD, (b) how cognitive impairment relates to different ARTs, and (c) whether machine learning could be used to predict genotype from SRS-2 subscales and items.

## Methods and materials

### Procedures

Participants included 350 individuals with one of seven GDDs, 171 typically developing (TD) controls, and 74 individuals with a behaviorally defined diagnosis of ASD. Absent any single, prospectively identified sample of youth with large cohorts of diverse GDDs and TD controls, the current sample was collated via collaborations across several research labs studying GDDs at the National Institutes of Health Intramural Research Program. Across IRB-approved protocols, informed consent was obtained from participants/guardians and study procedures adhered to guidelines set forth in the Declaration of Helsinki. In addition, an ASD sample was compiled from the National Database on Autism Research.

### Participants

The GDD sample consisted of 7 subgroups: (i) four groups with differing sex chromosome aneuploidies: “plusX” (XXY, XXX, *n* = 89^†^ (^†^A subset of the sex chromosome aneuploidy cohort was previously independently reported with regard to their SRS-2 total raw and *T*-scores [22])), “plusY” (XYY, *n* = 27^†^), “plusXX” (XXXY, XXXX, XXXXY, XXXXX^†^, *n* = 28), and “plusXY” (XXYY, *n* = 25), (ii) “DS” (Down syndrome, trisomy 21, *n* = 22), (iii) “WS” (Williams–Beuren syndrome, del7q11.23, *n* = 93^‡^ (^‡^A subset of the WS cohort was previously independently reported [23, 24].)), and (iv) “SMS” (Smith–Magenis syndrome, del17p11.2 or *RAI* mutation, *n* = 66). Genetic testing procedures for the GDD groups are provided in the Supplemental Material. Participant characteristics are summarized in Table 1.

**Table 1:** Demographics and SRS-2 total scores.

	Gene dosage disorder (GDD) groups															Benchmark groups				
	DS		PLUS X		WS		PLUS XX		PLUS XY		PLUS Y			SMS		ASD			TD	
	*N*	% ♂	*N*	% ♂	*N*	% ♂	*N*	% ♂	*N*	% ♂	*N*	% ♂		*N*	% ♂	*N*	% ♂		*N*	% ♂
Sample size & % Male_a_	22	63.6	89	62.9	93	47.3	28	82.1	25	100.0	27	100.0		66	40.9	74	55.0		171	79.7
	***M***	**SD**	***M***	**SD**	***M***	**SD**	***M***	**SD**	***M***	**SD**	***M***	**SD**		***M***	**SD**	***M***	**SD**		***M***	**SD**
	**(Range)**		**(Range)**		**(Range)**		**(Range)**		**(Range)**		**(Range)**			**(Range)**		**(Range)**			**(Range)**	
Age_b_	14.3	5.81	14.1	5.2	16.0	8.0	11.8	5.7	14.7	6.5	12.9	3.8		12.7	6.5	10.8	5.2		15.3	5.9
	(5–24)		(5–26)		(4–32)		(4–23)		(4–25)		(6–20)			(4–30)		(4–24)			(5–30)	
IQ Standard Score^1,2^_c_	49.6	17.2	96.2	14.7	60.9	13.1	65.6	9.5	85.8	12.5	92.5	18.3		55.7	10.6	85.4	23.8		115.7	13.5
	(24–92)		(61–134)		(42–85)		(51–81)		(68–119)		(60–141)			(40–80)		(33–140)			(83–142)	
Cognitive Impairment *T*-score^3^_d_	83.6	11.5	52.5	9.8	76.1	8.7	72.9	6.3	59.5	8.3	55.0	12.2		79.5	7.1	59.8	15.9		39.5	9.0
	(55–100)		(27–76)		(60–87)		(62–82)		(37–71)		(22–76)			(63–90)		(23–94)			(22–61)	
SRS Total_e_	60.2	10.8	61.3	12.6	64.7	11.0	65.1	10.0	66.9	8.8	68.7	13.3		73.8	10.2	78.2	11.0		45.4	6.5
	(46–80)		(38–89)		(40–90)		(49–83)		(52–85)		(43–89)			(56–93)		(47–102)			(36–77)	

1: IQ Standard Score: Mean = 50, SD = 10; Lower scores denote greater impairment; See the “Methods and materials” for details. 2: See supplemental Table 1 for details about IQ subsample including sample sizes for each group. 3: Cognitive Impairment *T*-score: Mean = 100, SD = 15; higher scores denote greater impairment; Note: These scores were created by transforming IQ standard scores to *T*-scores to be on the same scale as the SRS-2. a: Plus XX > WS, SMS; Plus XY > Plus X, WS, SMS; Plus Y > Plus X, WS, SMS. b: WS > SMS. c: DS < All except WS, SMS; Plus X > All except Plus X/Y, Plus Y; WS < all except DS, SMS, Plus 2/3X; Plus X/Y > All except Plus X, Plus Y; Plus Y > all except Plus X, Plus XY. d: DS > All except WS, SMS; Plus X < All except Plus X/Y, Plus Y; WS > all except DS, SMS, Plus 2/3X; Plus X/Y < All except Plus X, Plus Y; Plus Y < all except Plus X, Plus XY. e: SMS > All except Plus X/Y, Plus Y.

In the current investigation, we included males and females with an extra X chromosome in our ‘plusX’ and ‘plus XX’ groups. Combining male and female carriers of a supernumerary X-chromosome into a single “plus X” group was supported by prior behavioral studies of SCAs (e.g., refs. [22, 25]) and lack of statistical evidence for such an interaction in our own data (i.e., no statistically significant *T*-score differences between XXX and XXY groups).

### Measures

#### ART measurement

The SRS-2 [7] consists of 65 items and taps social functioning as well as restricted interests and repetitive behaviors. Parents reported on their child’s behavior using the Preschool (ages 2.5–4.5 years; *n* = 13), School-Age (ages 4–18 years; *n* = 498), or Adult (ages 19+; *n* = 87) forms. The Preschool and Adult forms of the SRS-2 were created, respectively, as downward and upward extensions of the original SRS, which had ages that correspond to the SRS-2 school age form. Of the 65 items on the SRS-2, 32 items are identical across the age versions; 33 items are adjusted due to differences in developmental expectations, with the majority only involving slight modifications to item content to fit with the relevant age group (e.g., referring to children vs. adults in the item or referring to ‘playing with’ rather than ‘interacting with’ peers). Thus, the SRS-2 is well-suited to describing clinical groups across a wide age range (see ref. [26] for an example of another study that used the SRS-2 in a similar manner).

The Preschool, School-Age, and Adult SRS-2 forms each consist of the same five treatment subscales which are derived by summing their constituent items across the three forms. Descriptions of the treatment subscales, including the number of items by subscale and example behaviors, are provided in Table 2. These subscales yield normative *T*-scores (i.e., age-group-normed [Preschool, School-Age, Adult] for all three SRS-2 forms and sex-normed for the SRS-2 School-Age form) which were used to compare ART scores across groups and to examine relations between ARTs and cognitive impairment. For machine learning analyses, raw SRS-2 subscale and item scores with age and sex covaried were used. The SRS-2 has strong psychometric characteristics, with high internal consistency (*α* > 0.90) and test-retest reliability (*r* ≥ 0.88).

**Table 2:** SRS-2 treatment subscales.

Subscale	Abbreviation	Items	Examples of behaviors
Social Awareness	Soc_Awr	8	E.g., unaware of others’ thoughts/feelings; doesn’t mind being out of sync with others
Social Cognition	Soc_Cog	12	E.g., difficulties extracting meaning from conversations; difficulty understanding the meaning of facial expressions, tone of voice
Social Communication	Soc_Com	22	E.g., difficulties expressing feelings; atypical eye contact
Social Motivation	Soc_Mot	11	E.g., prefers to be alone; needs to be told to join group activities
Restricted Interests and Repetitive Behavior	RIRB	12	E.g., difficulties with changes in routines; thinks/talks about the same topic repetitively

#### Cognitive impairment measurement

Because participants were enrolled in different investigations, several tests were used to estimate cognitive impairment (i.e., intellectual functioning). These are detailed in the Supplemental Material. For the current investigation, IQ standard scores (mean = 100; SD = 15) were transformed to *T*-scores that have the same polarity (higher scores = greater impairment) and distribution (mean = 50, SD = 15) as the SRS-2. Thus, they are described as *cognitive impairment* rather than IQ.

### Statistical analyses

Prior to running primary analyses, SRS-2 data were inspected and found to be normally distributed and free of outliers (>3 SDs from mean). Primary analyses were as follows.

#### Evaluation of SRS-2 profiles among the GDD groups

To examine SRS-2 profiles using normative *T*-scores, a 7 (GDD groups) × 5 (SRS-2 subscale) mixed-model ANOVA was completed (followed by an ANCOVA with cognitive impairment covaried). Then two complementary approaches were used to provide finer-grained descriptions of group and subscale effects. First, a series of ANOVAs (and ANCOVAs with cognitive impairment covaried) were completed to test the effect of GDD on each SRS-2 subscale and the effect of SRS-2 subscale within each GDD (see Table 3). Multiple comparisons were controlled with a Bonferroni-correction for the number of SRS-2 subscales (*p* = 0.01 [0.05/5]) and the number of GDDs (*p* = 0.007 [0.05/7]).

**Table 3:** Age and sex normed *T*-scores on the SRS-2 by GDD and scale.

	DS		PLUS X		WS		PLUS XX		PLUS XY		PLUS Y		SMS		Group effect		Group effect - Cog Imp Cov.	
	*M*	SD	*M*	SD	*M*	SD	*M*	SD	*M*	SD	*M*	SD	*M*	SD	*F*	*η*_p_^2^	*F*	*η*_p_^2^
SRS Social Awareness	61.0	9.2	57.6	12.1	61.9	10.5	61.3	10.0	65.7	10.4	66.9	11.8	71.8	9.0	12.5^a^	0.18	4.9^a^	0.13
SRS Social Cognition	64.6	12.5	62.6	12.9	69.8	12.0	65.7	8.7	68.0	10.5	65.8	13.0	72.7	9.6	5.9^a^	0.09	2.7	0.07
SRS Social Communication	59.4	11.2	61.5	12.8	62.1	10.7	65.0	10.6	66.4	9.7	68.9	14.4	71.5	11.1	7.1^a^	0.11	4.5^a^	0.12
SRS Social Motivation	51.8	9.6	58.9	12.6	52.9	8.8	60.1	9.3	58.8	10.3	63.7	11.7	62.3	11.0	8.2^a^	0.13	5.7^a^	0.14
SRS RIRB	58.9	12.5	58.1	10.9	68.4	12.4	63.8	10.7	66.3	10.7	66.0	13.0	77.6	11.7	19.9^a^	0.26	7.4^a^	0.18
	***F***	***η***_*p*_^2^	***F***	***η***_p_^2^	***F***	***η***_p_^2^	***F***	***η***_*p*_^2^	***F***	***η***_p_^2^	***F***	***η***_p_^2^	***F***	**η**_**p**_^**2**^				
Scale effect	10.6^a^	0.34	10.6^a^	0.11	117.5^a^	0.56	5.4^a^	0.17	6.2^a^	0.21	2.3	0.08	43.5^a^	0.40				

^a^*p* < 0.05; survives Bonferroni-correction for multiple comparisons; see “Statistical analysis” section for further details.

**Fig. 1:**
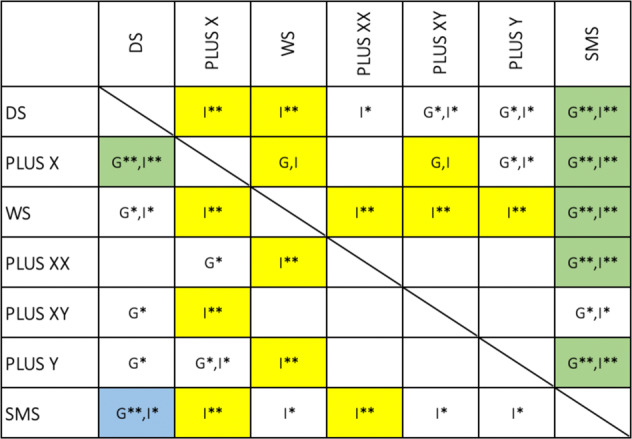
Synopsis of results from pairwise mixed model ANOVAs (above diagonal) and ANCOVAs with cognitive impairment covaried (below diagonal). To test for differential ART subscale profiles between each unique pair of GDD groups, we ran 21 (number of unique GDD group pairings) 2 (GDD group) × 5 (SRS-2 subscale) mixed-model ANOVAs. For these analyses, group effects (e.g., GDD 1 is more or less impaired overall than GDD 2 on the SRS-2 subscales) and group*subscale interaction effects (e.g., there is a difference in the SRS-2 profile for GDD 1 vs. GDD 2) were evaluated and results are presented above the diagonal. Parallel analyses were also completed using ANCOVA including cognitive impairment as a covariate and results are presented below the diagonal. When interpreting the figure, note the following. Main effects of group were denoted with a ‘G’; group*scale interactions were denoted with an ‘I’. Instances in which there was a main effect of group or group*scale interaction that did not survive Bonferroni correction are denoted with a single asterisk (*p < 0.05); those that survived Bonferroni correction are denoted with a double asterisk (**p < 0.05—Bonferroni corrected). Finally, to aid interpretation, color coding was implemented as follows. When a main effect of group was identified that survived Bonferroni correction, the cell in the matrix was color coded *blue*. When there was a Bonferroni-corrected group*subscale interaction, the cell was color coded *yellow*. Instances in which there was both a main effect of group (magnitude of impairment) and a group* subscale interaction (SRS-2 profile difference) following Bonferroni correction were color coded *green*.

Second, SRS-2 profile differences (i.e., differences in the pattern of test scores/strengths and weakness) were evaluated using a series of mixed-model ANOVAs. Specifically, 21 mixed model ANOVAs (each consisting of one between-subjects factor [group: GDD1 vs. GDD2] and one within-subjects factor [SRS-2 subscale]) were run. For these analyses, group effects (e.g., GDD1 is more/less impaired than GDD 2 overall) and group*subscale interaction effects (e.g., GDD1 and GDD2 have different profiles) were evaluated. To adjust for the number of effects examined (21 group effects, 21 group*subscale interactions), statistical significance was evaluated against a Bonferroni-corrected *p*-value of *p* < 0.001 (=0.05/42) (Fig. 1, upper triangle). These analyses were re-run with cognitive impairment covaried (Fig. 1, lower triangle).

**Fig. 2:**
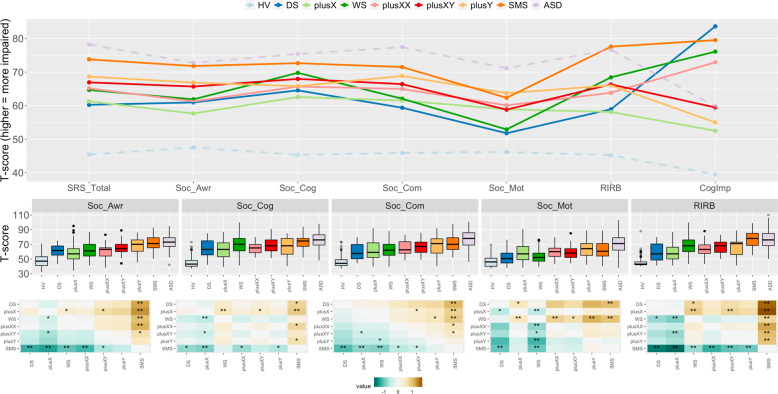
Gene dosage disorder (GDD) scores for different autism-related traits (ARTs). *Top panel:* Point-line graph showing score profiles for each GDD across ARTs. Color encodes group. GDDs are in solid lines, and the benchmark autism spectrum disorder (ASD) and healthy volunteer (HV) groups are in dashed lines. *Middle panel*: Boxplots for each ART showing GDD group score distributions. *Bottom panel*: Heatmaps for each ART showing Cohen’s *d* effect sizes for all pairwise GDD group comparisons (column group vs. row group). Asterisks denote statistically significant comparisons (*nominal *p* < 0.05, **surviving Bonferroni correction for multiple comparisons).

**Fig. 3:**
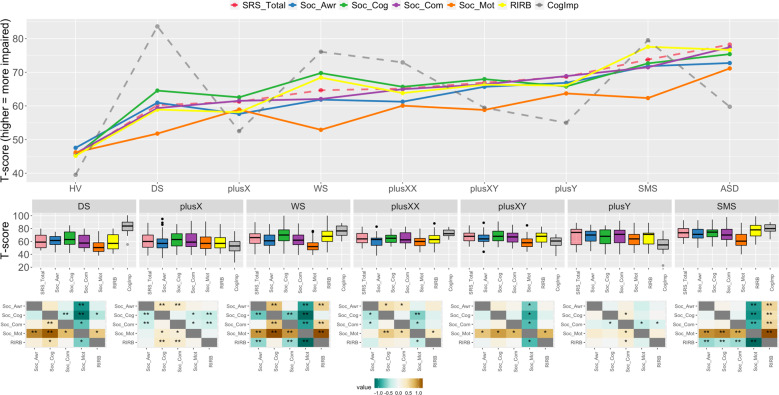
Autism-related trait (ART) scores for different gene dosage disorders (GDDs). *Top panel:* Point-line graph showing score profiles for each ART across GDDs. Colored solid lines encode ARTs. Cognitive impairment scores are shown as a reference (dashed gray). *Middle panel*: Boxplots for each GDD showing score distributions for each ART. *Bottom panel*: Heatmaps for each GDD showing Cohen’s *d* effect sizes for all pairwise ART comparisons (column group vs. row group). Asterisks denote statistically significant comparisons (*nominal *p* < 0.05, **surviving Bonferroni correction for multiple comparisons).

In order to interpret the group*subscale interactions that indicated a profile difference between different GDD pairs, ANOVAs/ANCOVAs yielding a significant group*subscale interaction were followed up with post-hoc comparisons between (ANOVAs: Bonferroni-corrected *p* = 0.0008 [=0.05/60]; ANCOVAs: corrected *p* = 0.001 [=0.05/35]) and within (ANOVAs and ANCOVAs: Bonferroni-corrected *p* = 0.0007 [=0.05/70]) groups, see Fig. 2 (between-group) and Fig. 3 (within-group). Note that all *p*-values reported in the manuscript in which group means were compared are two-tailed tests.

#### Relationship between ARTs and cognitive impairment across GDD groups

**Fig. 4:**
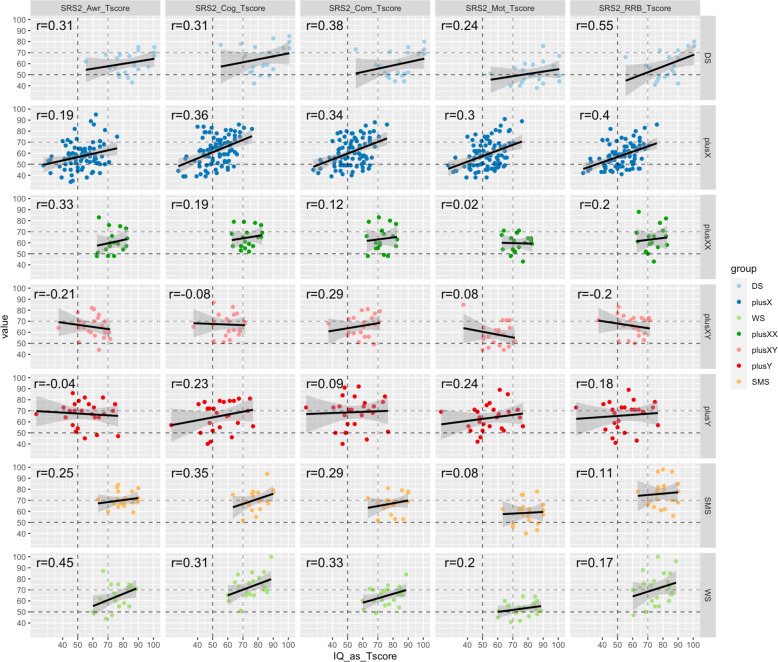
Relationships between autism-related trait (ART) scores and cognitive impairment within each gene dosage disorder (GDD). Scatterplots and linear fit lines showing the relationship between increasing cognitive impairment (*x*-axis: “IQ_as_Tscore”) and ART score value (*y*-axis) faceted by GDD (rows) and ART subscale (columns). Robust correlation coefficients are provided for each cell. Color encodes GDD. Dashed lines show population norm values (50, black) and 2 standard deviations above this norm (70, gray). Note that IQ is inverted and transformed to a distribution with mean = 50, sd = 10 to form “IQ_as_Tscore” (i.e., IQ_as_Tscore > 70 is equivalent to IQ < 70).

Linear mixed models were used to test for variation in ART–cognitive impairment relationships as a function of both GDD group and SRS-2 subscale. Models with successively lower-order interactions between the fixed effects of GDD group, ART subscale, and cognitive impairment were compared using ANOVAs, with participant ID as a random effect. Figure 4 visualizes these relationships. For each unique cell in this matrix, a linear relationship between cognitive impairment and ARTs was quantified using percentage bend correlation (selected for robustness to outliers) as implemented in the R package *correlations*.

#### Prediction of genotype from ARTs

To examine whether ART profiles across GDDs are sufficiently distinctive that they can be used to predict genotype from SRS-2 ratings, machine learning techniques including least absolute shrinkage and selection operator (LASSO) [27] and group LASSO [28] were used. These models have been widely applied and have been shown to yield high prediction accuracy and robust pattern identification [29–32].

LASSO was conducted to examine whether each of the GDD groups can be distinguished from the remaining groups’ data utilizing the five SRS-2 subscales. For each of the 7 GDD groups (i.e., the target group), the remaining data were constructed to be balanced and representative by randomly sampling subjects from each of the other 6 GDD groups with equal sizes. The resulting mixed-GDD group was required to have the same sample size as the target group. This allowed balanced data between the target group and the mixed-GDD group as suggested by the machine learning literature [33, 34].

Group LASSO, selected to account for potentially high correlations among the items, was also applied to test how well each of the GDD groups can be differentiated from all others using the 65 SRS-2 items. To evaluate whether the predictive ability of the SRS-2 subscales or items varied by including cognitive impairment in the model, we re-ran the LASSO and group LASSO models with cognitive impairment covaried.

**Fig. 5:**
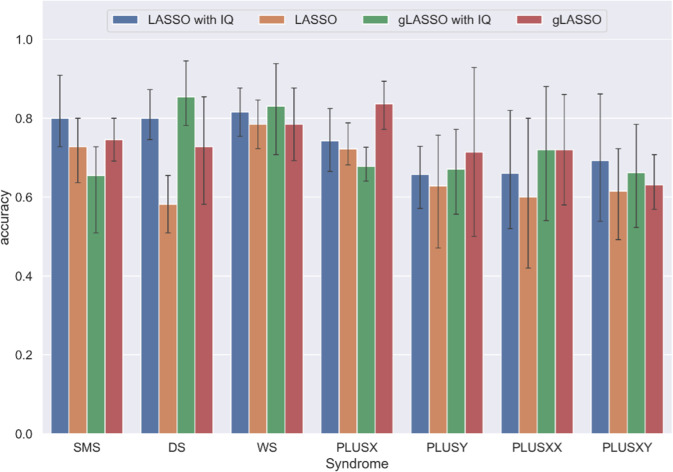
Average prediction accuracy for each GDD group achieved by each of the four machine learning (ML) models. All the models yield plausible (above chance) prediction accuracy. Without IQ, group LASSO with 65 items performs better than LASSO with 5 subscales for most of the GDD groups except WS where their prediction accuracies are similar. With IQ information, the comparison of ML model performance with 65 items vs. 5 subscales varies across the GDD groups. The error bar indicates 95% confidence interval of the 1000 bootstrap samples across the 5-fold cross-validation.

**Fig. 6:**
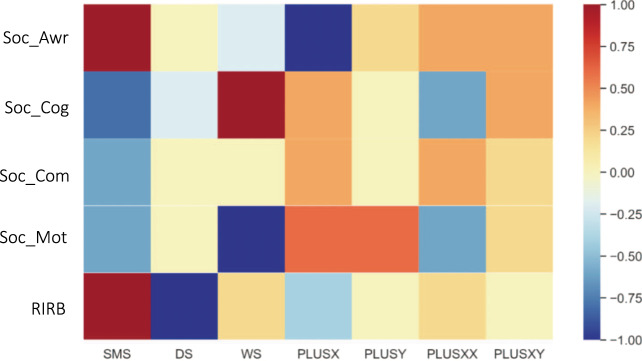
Feature importance for the LASSO model with IQ included. The coloring represents both the feature importance and the direction of feature effect. Positive values indicate an increased likelihood of a certain GDD group while negative values suggest a reduced probability of a certain GDD group. Higher absolute values (i.e., feature importance values) indicate greater consistency of a feature being selected as an important predictor to improve prediction accuracy across the 5-fold cross-validation. Awareness is consistently selected as an important predictor for SMS and PLUSX. Cognition is consistently selected for predicting SMS and WS. Motivation is consistently selected for predicting WS, PLUSX, and PLUSY. RIRB is consistently selected for predicting SMS and DS.

For each model, a nested 5-fold cross-validation (CV) was used to tune the model penalty parameter and evaluate model performance. Prediction accuracy was averaged across the 5-fold CV, and each model’s 95% confidence interval was reported (Fig. 5). A heat map was created (Fig. 6) for the most parsimonious model (with cognitive impairment covaried) to visualize feature importance and the direction of the feature effect (e.g., a positive or negative effect). The feature importance was calculated as the proportion of times that a feature was selected by a machine learning model as an important predictor to improve prediction accuracy across the 5-fold CV.

Supplemental Table 2 summarizes the analytic plan.

## Results

### SRS-2 profiles across and within GDDs

Table 3 details the mean SRS-2 subscale scores by group. All were elevated above the normative reference *T*-score of 50 (*p*s < 0.004), with the exception of the Soc_Mot subscale in DS. Mixed model ANOVA revealed main effects of GDD group (*F* [6,343] = 10.26, *p* < 0.001, *n*_p_^2^ = 0.15) and SRS-2 subscale (*F*[4,1215] = 68.90, *p* < 0.001, *n*_p_^2^ = 0.17); these main effects were qualified by a significant group*subscale interaction (*F*[21,1215] = 12.18, *p* < 0.001, *n*_p_^2^ = 0.18), indicating that SRS-2 *profiles* differed among groups. A follow-up ANCOVA with cognitive impairment covaried also identified a statistically-significant main effect of group (*F*[6, 207] = 5.00, *p* < 0.001, *n*_p_^2^ = 0.13), qualified by a statistically significant group*subscale interaction (*F*[21, 716] = 5.04, *p* < 0.001, *n*_p_^2^ = 0.13). Thus, there were differences in SRS-2 profiles among GDD groups that could not be fully explained by variation in cognitive impairment.

The effects of GDD group on each subscale and of subscale within each GDD group were next evaluated with univariate ANOVA (see Table 3) (see Supplemental Table 3 for estimated marginal means/effect sizes estimates with cognitive impairment held constant). After Bonferroni-correction, significant effects of group were observed for all subscales (except Soc_Cog with cognitive impairment covaried), indicating significant variation in ARTs across GDDs. The proportion of variance explained by GDD group differed between ART subscales, with a high of 26% (RIRB) and a low of 9% (Soc_Cog). Statistically significant within-group variation across ART subscales was also observed for all GDDs, except the +Y group.

Next, as is common practice in neurodevelopmental disorders research [22, 35–37], SRS-2 profile differences (i.e., differences in the pattern of test scores/strengths and weakness) were evaluated using a series of mixed-model ANOVAs (or ANCOVAs covarying for cognitive impairment). Specifically, 21 mixed model ANOVAs/ANCOVAs (each consisting of one between-subjects factor [group: GDD1 vs. GDD2] and one within-subjects factor [SRS-2 subscale]) were completed. For these analyses, group effects indicating an overall difference in the magnitude of impairment between two groups (e.g., GDD1 is more/less impaired than GDD 2 overall) and group*subscale interaction effects (e.g., GDD1 and GDD2 have a different pattern of scores on the SRS-2 subscales) were evaluated. The results of these ANOVAs and ANCOVAs are presented in Fig. 1 (upper and lower triangles, respectively). As seen in Fig. 1, when a main effect of group (i.e., a difference between the two GDDs being compared in the overall magnitude of impairment) was identified, the cell in the matrix was color coded blue. When there was a group*subscale interaction (i.e., the profile or pattern of SRS-2 subscale scores differed between the two GDDs being considered), the cell was color coded yellow. Lastly, instances in which there was both a main effect of group (magnitude of impairment) and a group*subscale interaction (SRS-2 profile difference) were color coded green. As evidenced by the preponderance of yellow cells in the matrix, the most common form of between-group ART differences was for the *profile* of ART scores in the absence of group differences in overall ART severity, regardless of whether cognitive impairment was covaried. The GDD groups that most differed from others in their SRS-2 profiles were the WS and plusX groups. The SMS group was notable for often showing differences in overall SRS-2 scores and profile.

The data underlying the statistical comparisons detailed above are presented visually in Figs. 2 and 3. For those pairwise GDD comparisons characterized by group*subscale interactions, follow-up pairwise *t*-tests were completed to identify which SRS-2 subscales differed between and within GDD groups (see bottom panels of Figs. 2 and 3, respectively). For results with cognitive impairment covaried, see Supplementary Table 2.

### ART–IQ relationships across GDDs and SRS-2 subscales

Analysis of variance comparisons of nested mixed models failed to find evidence that relationships between SRS-2 scores and cognitive impairment are significantly modulated by interactive effects of GDD and ART subscale (*p* = 0.57). However, the relationship between cognitive impairment and ART scores did vary significantly as a function of subscale (controlling for a main effect of GDD group, *p* < 10^−10^). Thus, different SRS-2 subscales vary from each other in the nature of their relationships with cognitive impairment, but this does not differ significantly across GDD groups.

Given these results, standardized regression coefficients were estimated for IQ as a predictor of each SRS-2 score while controlling for the main effect of GDD group. This revealed that greater cognitive impairment was associated with more severe ARTs for all subscales, but that the magnitude of this relationship (i.e., regression slope, *β*) varied by ART subscale: Soc_Cog (*β* = 0.3, *p* = 0.000002), Soc_Com (*β* = 0.28, *p* = 0.00002), RIRB (*β* = 0.26, *p* = 0.00005), Soc_Mot (*β* = 0.24, *p* = 0.0003), Soc_Awr (*β* = 0.17, *p* = 0.01). Although GDD group did not significantly modulate the relationship between the SRS-2 subscales and cognitive impairment, scatterplots (with robust correlation coefficients) of the relationship between each SRS-2 subscale and cognitive impairment are provided for each GDD group (Fig. 4), given the rarity of these conditions.

### Prediction of genotype from SRS-2 scores

Finally, machine learning was used to assess if SRS-2 score variation across GDDs was sufficient to predict an individual’s GDD grouping. Models included either the five SRS-2 subscales (LASSO) or the 65 items (group LASSO) and were run with and without cognitive impairment covaried. Figure 5 displays prediction accuracy for the four models. Average prediction accuracy was similar across models, with most models having 60–80% prediction accuracy. As the magnitude of the differences between the models was modest, we focus on the results of the LASSO model (utilizing the 5 subscales) with cognitive impairment covaried for parsimony’s sake in order to highlight which model features (i.e., SRS-2 subscales) were of the greatest importance when cognitive impairment was held constant. To visualize, a heatmap was created (Fig. 6) that depicts feature-importance for predicting group membership. Higher absolute feature-importance values indicated greater consistency of a feature being selected as an important predictor across the 5-fold CV. To simplify interpretation, features with an importance value >0.75 were considered to be consistently selected by LASSO. The maximally important predictive features varied between GDDs, with notably important SRS-2 subscale predictors including: Soc_Mot for WS, plusX, and plusY; Soc_Awr for SMS and plusX; RIRB for SMS and DS; and Cognition for SMS and WS. The Soc_Com subscale was notably of limited importance for predictive accuracy of all GDDs.

## Discussion

The analyses presented in this report detail the profile of ARTs within and across 7 GDDs and provide fresh insights into how ART profiles vary across these genetically defined groups. First, our study replicates and adds to prior single-disorder reports of ARTs in the specific GDDs considered. For example, consistent with studies that examine diagnoses of ASD among different GDDs, our continuous examination of ARTs revealed the lowest impairment in the DS group [38]. In contrast, the SMS group presented with highest ART impairment, particularly in the realm of repetitive behavior, consistent with past reports [39, 40]. Within the WS group, social cognition was a peak impairment (along with repetitive behavior), whereas social motivation was largely preserved, consistent with prior research [41]. Lastly, within the sex chromosome aneuploidy subgroups, elevated ARTs were observed relative to normative expectations. Moreover, ARTs were nominally more impaired in those with an extra Y compared to those with an extra X, consistent with prior research [35].

By directly comparing the GDDs studied, we document considerable variegation in ART profiles as a function of GDD that is largely maintained when controlling for cognitive impairment. For example, without controlling for cognitive impairment, we observed 12 unique pairwise GDD group differences in ART profiles. Six of these differences were with WS and appeared to be driven by the relative preservation of social motivation in this group. Other notable profile differences include the relative preservation of social motivation in DS as compared to SMS, and greater severity of many ARTs in SMS as compared to several other GDDs. Seven unique pairwise GDD group differences in ART elevation profiles were apparent after controlling for cognitive impairment. Salient aspects of this variegation above and beyond cognitive impairment included the relative preservation of social motivation in WS and the relatively low level of RIRBs in plusX. Consistent with prior research, these findings suggest that there are meaningful differences in the profile of ART elevations (i.e., the pattern of scores on the different scales as opposed to the overall severity of ART elevation) between different GDDs [5].

In further support of this notion, we find evidence for the discriminability of GDDs by ART profile from multivariate machine learning analyses. In particular, we found moderate to high levels of prediction for most of the GDD groups using sparse regression models which varied as a function of granularity of features examined (5 subscales vs. 65 items) and whether cognitive impairment was included in the model. Overall, a comparison of models suggested similar levels of prediction accuracy for models in which the 5 subscales or 65 items were used as predictors. For parsimony, we focus on the 5 subscale model in which cognitive impairment was covaried. From this model, we learned the following: the maximally important predictive features vary between GDDs, with notably important SRS-2 subscale predictors including Soc_Mot for all GDDs except SMS and plusXY, Soc_Awr for SMS and DS, RIRB for SMS, DS, and WS, and Soc_Cog for WS. The Soc_Com subscale was of limited importance for predictive accuracy in all GDDs. One possible explanation for this is that all of the disorders studied are characterized by some degree of language impairment [42–45]. Even impairments in non-social facets of language are likely to impact social communication abilities by limiting the toolkit needed to effectively communicate. Thus, this ART may carry a lower level of specificity across different GDDs.

Our findings carry implications for both basic and clinical neuroscience. The observation that different GDDs can induce different ART profiles suggests that the human brain systems underlying different ARTs must be dissociable at some level. For example, disruptions of the different gene sets that define each GDD may achieve dissociable changes in social motivation as compared to repetitive behavior by altering the development of different features of the brain [46]. However, our findings also indicate that some ART elevations appear to show less variability across GDDs, suggesting that the genetic fractionability of these traits may be lower than that of other ARTs. Thus, there may be many more routes to impacting highly integrative brain outputs than there are for impacting more granular aspects of behavior such as the tendency to show restricted and repetitive behaviors. A goal for future work will be testing if traits that are highly variegated across GDDs also show distinct brain-behavior correlations within GDDs.

Dissociability of ART profiles across GDDs is also important from clinical and translational perspectives. If a clinically relevant trait is highly differentiated across GDDs, knowing an individual’s GDD subtype could help to tailor care. Of the ARTs, patterns of RIRBs may most closely fit this scenario. Given the significant functional impact of these behaviors [47] this may be a priority area for attempted tailoring of clinical care to GDD. Although on a longer timeframe, our findings also inform prospects for mechanistically informed treatments for ARTs. Specifically, the non-specific elevation of a certain ARTs across multiple GDDs implies shared mechanistic pathways that, if successfully targeted, could provide a generalizable path to cross-GDD interventions.

Our findings should be considered in light of several limitations. First, we examine ARTs in the absence of diagnostic information about ASD or other psychiatric disorders. However, there is extensive evidence for continuity between continuous measures of ARTs and categorical ASD diagnoses [48]. Also, ART elevation is well-recognized across many non-ASD psychiatric diagnoses [9–11], and ASD itself is often comorbid with other psychiatric diagnoses [49]. Taken together, we believe these considerations support our focus on dimensional ARTs. Second, the key analytic outcomes of our study design may vary with overall severity of clinical impairment and/or participant age. In this context, the potential for ascertainment bias and our inability to meaningfully model age effects represent notable limitations. However, these are broader challenges for neuropsychiatric research in rare disorders and will only be overcome with detailed dimensional data on large, longitudinal and population-based GDD cohorts (e.g., ref. [50]). Such data will enable our field to address critical open questions including the clinically important potential for developmentally dynamic shifts in ARTs, which may themselves vary between different GDDs. A related challenge in seeking to understand these developmental dynamisms is the tension between needing instruments that capture age-specific symptomatology while also generating output metrics that can be combined across different age ranges and are scaled relative to age expectations. This tension is reflected in need to use the Preschool, School-Age, and Adult forms of the SRS-2 in the current research. This limitation in our method represents a common a challenge faced by researchers studying neurodevelopmental disorders—i.e., the limited number of assessment tools that are available to evaluate cognition and behavior for individuals with a wide range of chronological and/or mental ages (for a review, see ref. [51]). Third, smaller sample sizes in some GDD groups impacted the ability to detect statistically significant differences between and within groups. We addressed this by also providing effect size estimates. However, future research could benefit from studying GDD groups with equal sample sizes. Fourth, the current study’s sample was compiled across multiple research labs and was not prospectively ascertained. Although this may be conceptualized as a sample of ‘convenience,’ our approach permitted comparing diverse GDDs, both in term of genotypic variation and behavioral presentation. We hope that this will encourage future research in which prospectively identified samples of youth with diverse GDDs may be studied to further elucidate the GDD-specific and shared ARTs that characterize these unique groups. Lastly, the SRS-2 has been critiqued for both its lack of statistically derived factor structure and differential measurement in phenotypically diverse populations [52], such as high and low IQ. Although this criticism should be considered when interpreting the current study’s findings, it is important to note that the SRS-2 is one of the best tools available to measure continuous autistic traits in large samples of participants. Acknowledging this limitation, we hope that the current study will spur further research with diverse GDD samples using alternative tools and assessment approaches with the goal of further distilling GDD-specific and shared traits. It is our hope that such research will inform both basic and clinical science and ultimately support quality of life for individuals with GDDs.

## Supplementary information

Supplemental text
