# Supplementary material for: Variegation of autism related traits across seven neurogenetic disorders
Source: Transl Psychiatry. 2022 Apr 7;12:149. doi: 10.1038/s41398-022-01895-0 (PMC8989950; doi:10.1038/s41398-022-01895-0)

**SUPPLEMENTAL MATERIAL**

**Methods & Materials**

***Participants***

Genetic testing procedures. For participants with sex chromosome aneuploidies, presence of the specified genetic disorder was confirmed at initial enrollment by karyotypic analyses. For participants with DS, karyotype confirmation of trisomy 21 was confirmed in 82% of the cases. 18% refused the blood draw needed for karyotype analysis, but parents reported a confirmed genetic diagnosis of Down syndrome via earlier testing. Individuals with WS had genetic testing at admission to the study that showed them to have (at minimum) deletion of the elastin gene, meaning that a clinical or research testing showed them to have only one copy of this gene, equivalent to the clinical fluorescent in situ hybridization (FISH) test. A subset of individuals also had clinical or research chromosome microarray, exome or genome sequencing--participants with known atypical deletion were excluded from this analysis (52). Individuals with SMS had either a cytogenetically confirmed deletion by FISH (del 17p11.2, n=58) or heterozygous RAI1 mutation (n=8) documented by DNA-based molecular testing via targeted gene panel and/or RAI1 (exon 3) sequence analysis. All participants with ASD drawn from the NDAR database for the current study met criteria for ASD on the developmentally appropriate version of the Autism Diagnostic Observation Schedule (ADOS) administered for their respective study. Participants included in the typically developing group were screened by phone prior to study entry to exclude for learning, psychiatric, developmental, and neurological disorders.

***Measures***

IQ Testing. Because the participants in the current sample were part of studies with different research foci, not all participants had estimated intellectual ability levels. IQ test results were available for 75% of the sample. **Supplemental Table 1** provides information about participants with and without IQ data, who showed no statistically-significant demographic differences. The following measures were used to characterize cognitive function: Differential Ability Scales – Second edition (School age: n=36; Early years: n=35); Kaufman Brief Intelligence Test- Second Edition (n=14); WAIS-IV (n=14); WASI (n=304); WISC-III (n=3); WISC-R (n=7); WISC-V (n=4); WPPSI-III (n=6). In addition, there were 26 participants with IQ data (provided by parents) but for whom test details were unavailable. Lastly, there were 147 participants without any IQ data available.


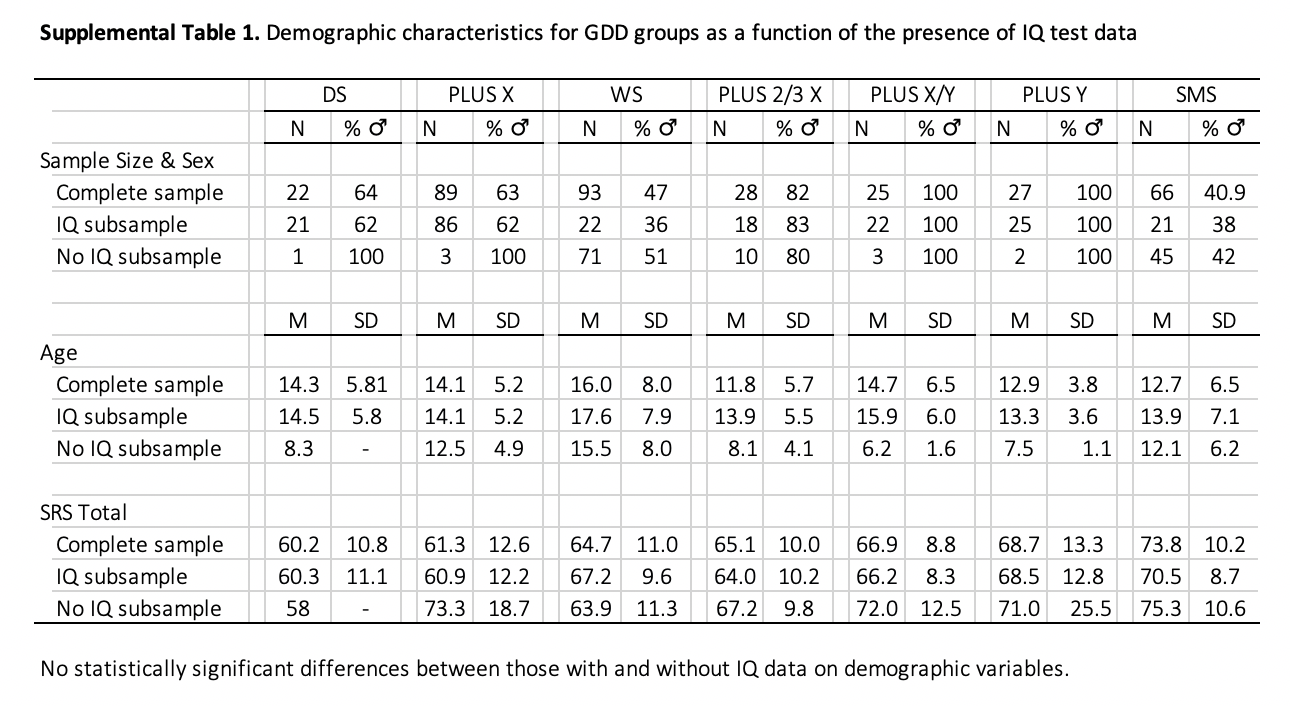

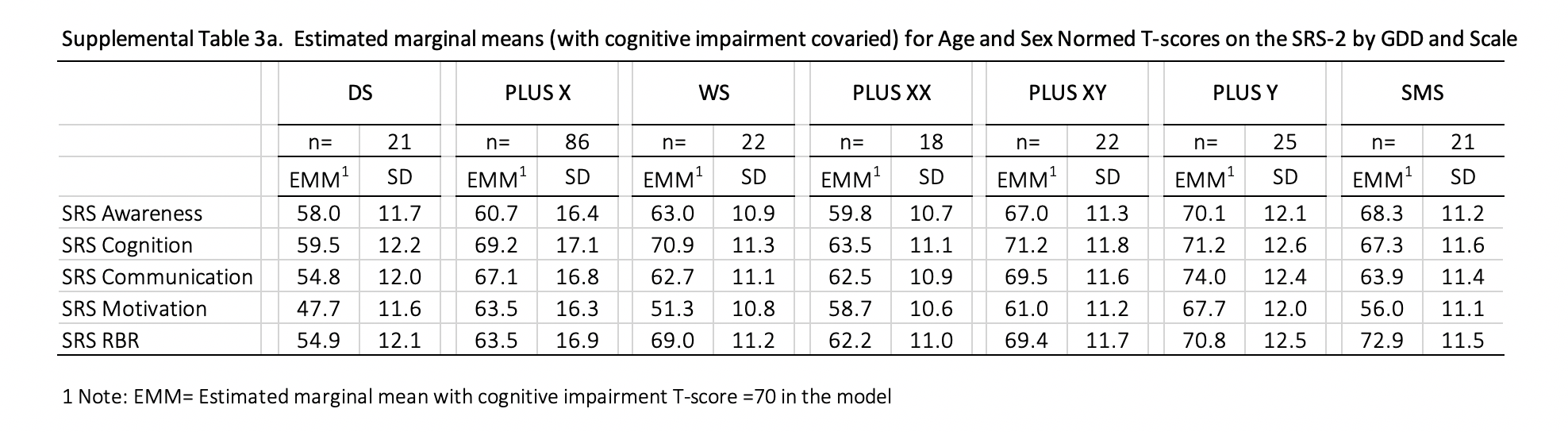


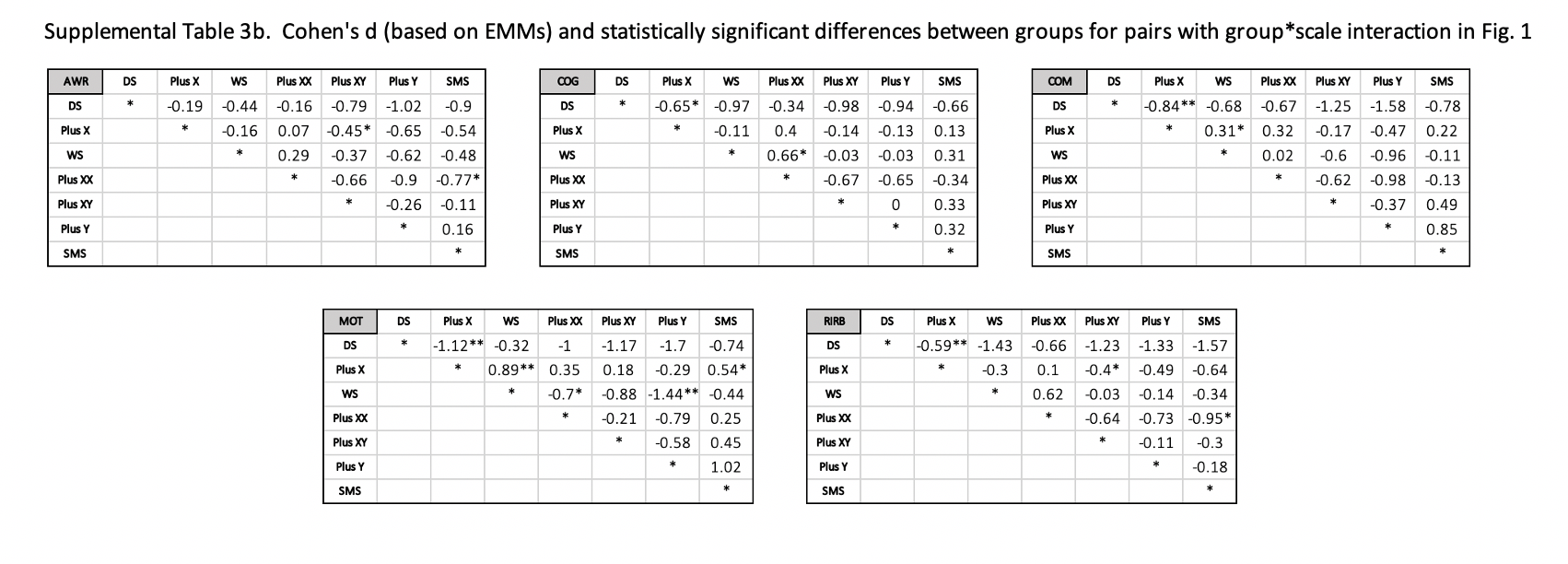

Supplement: Supplementary file 1 — Supplemental text [file 41398_2022_1895_MOESM1_ESM.docx]
